# *Saccharomyces cerevisiae* supplemented diets mitigate the effects of waterborne cadmium toxicity on gilthead seabream (*Sparus aurata* L.): growth performance, haemato-biochemical, stress biomarkers, and histopathological investigations

**DOI:** 10.1007/s11259-023-10176-0

**Published:** 2023-08-02

**Authors:** Mohsen Abdel-Tawwab, Riad H. Khalil, Nehal A. Younis, Talal A. M. Abo Selema, Adel H. Saad, Suzan O. M. El-Werwary, Ali H. Gouda, Ashraf M. Soliman, Sherien H. H. Shady, Mohamed N. Monier

**Affiliations:** 1 Department of Fish Biology and Ecology, Central Laboratory for Aquaculture Research, Agriculture Research Center, Abbassa, Abo-Hammad, Sharqia 44662 Egypt; 2 Department of Poultry and Fish Diseases, Faculty of Veterinary Medicine, Alexandria University, Alexandria, Egypt; 3 Department of Aquatic Animal Medicine and Management, Faculty of Veterinary Medicine, Cairo University, Cairo, Egypt; 4 Department of Nutrition and Clinical Nutrition, Faculty of Veterinary Medicine, Matrouh University, Matrouh, Egypt; 5 Department of Fish Hatching and Physiology, Central Laboratory for Aquaculture Research, Agriculture Research Center, Abbassa, Abo-Hammad, Sharqia 44662 Egypt; 6 Central Laboratory for Aquaculture Research, Sakha Aquaculture Research Unit, Kafrelsheikh, Egypt; 7 Limnology Department, Central Laboratory for Aquaculture Research, Agriculture Research Center, Abbassa, Abo-Hammad, Sharqia 44662 Egypt

**Keywords:** Dietary yeast, Cadmium toxicity, Gilthead seabream, Growth performance, Welfare status, Histopathological investigations

## Abstract

Yeast, *Saccharomyces cerevisiae*, has been utilized as a probiotic in aqua-feeds to promote growth and alleviate the stress in aquatic animals. On the other hand, cadmium (Cd) toxicity causes serious retardation of growth and welfare status of aquatic animals. The present study was conducted to evaluate the protective role of dietary yeast in mitigating the waterborne Cd toxicity effects on the growth, haemato-biochemical, stress biomarkers, and histopathological investigations of gilthead seabream (*Sparus aurata* L.). In a 3 × 3 factorial design, the acclimated fish (20–24 g) were randomly distributed into nine treatments in triplicates where they were fed on 0.0% (control), 0.5%, and 1.0% of yeast along with exposure to 0.0, 1.0, and 2.0 mg Cd/L for 60 days. All growth parameters and mRNA expressions of *IGF-1* and *GH* genes as well as haematological parameters were markedly increased with the increase of dietary yeast levels; meanwhile these variables were significantly retarded with Cd exposure. Contradictory effects on the above-mentioned variables were observed with Cd toxicity. In contrast, blood cortisol, glucose, total cholesterol, and triglyceride, lactate dehydrogenase, alanine transaminase, aspartate transaminase, alkaline phosphatase, in addition to DNA fragments % were noticeably increased with Cd toxicity especially at the treatment of 2.0 mg Cd/L, while decreasing with increasing dietary yeast levels. Compared with the control fish group, Cd concentrations in the gill, liver, and muscle tissues of gilthead seabream were higher in Cd-exposed treatments, especially at the treatment of 2.0 mg Cd/L. Deposition of Cd in fish liver was higher than that in gill tissues but lowest Cd residue was observed in muscle tissues. No significant changes in Cd residues in fish organs were observed in yeast-fed fish with no Cd exposure. The Cd exposure negatively affected histological status of gill, liver, and kidney tissues of *S. aurata*; while feeding Cd-exposed fish on yeast diets lowered the Cd residues in fish organs and recovered the adverse effects of Cd toxicity. Hence, this study recommends the addition of bakery yeast (1.0%) to fish diets to improve the performance, overall welfare, and histopathological status of gilthead seabream, *S. aurata*.

## Introduction

Heavy metals pollution including cadmium (Cd) comes from acid-mine drainage, industrial emissions, transportation, sewage, storm-water, air deposition, and construction materials (Xia et al. 2011) and they may reach aquatic ecosystems. Cadmium (Cd) is a heavy metal with high toxicity and the enduring ability for bio-magnification, bio-accumulation, and combination into the food chain (Ahmed et al. 2015; Kibria et al. 2016). Excessive bioaccumulation of Cd occurs in fish via its ingestion, ion exchange through gill and membrane surfaces, and adsorption by fish tissues from the aquatic ecosystem (Ahmed et al. 2014). It could cause biochemical, physiological, and oxidative stress abnormalities, anemia, and acute histopathological damage, among others (Abdel-Tawwab and Wafeek 2010, 2014, 2017; Al-Asgah et al. 2015; Otludil et al. 2017). In addition, Cd negatively impacts the survival, growth, and reproduction of several fish species (Kim et al. 2004; Szczerbik et al. 2006; Zhou et al. 2017; Elgendy et al. 2023). Therefore, there is an urgent need for low-cost, durable, and efficient strategies to reduce its toxicity effects on fish welfare. Accordingly, there is a growing interest in finding new substances or commercially accessible dietary supplements to enhance the growth and welfare of fish.

Specifically, yeast (*Saccharomyces cerevisiae*) is a fungus with single eukaryotic cells that contains 45% protein, 1% lipid, and 2.7% crude fiber (Solomon et al. 2017). Additionally, it contains considerable amounts of enzymes, oligosaccharides, amino acids, organic acids, vitamins, and minerals among others; thus, yeast (whole or fractions) are a popular product to use as supplements in aqua-feeds (Hansen et al. 2019; Rawling et al. 2019; Vidakovic et al. 2020; Agboola et al. 2021; Ceseña et al. 2021). The inclusion of yeast and/or yeast products in aqua-feeds have been used with nutritional and functional benefits on the growth, immune functions, and gut welfare in aquatic animals (Agboola et al. 2021; Ceseña et al. 2021).

Gilthead sea bream (*Sparus aurata* L.) is commonly cultivated in marine cages and recirculating aquaculture systems and it represents one of the most important marine finfish species reared in the Mediterranean region with an annual world production of approximately 160,000 tons (FAO 2019). Its aquaculture increased day by day in coastal Mediterranean regions; however, Cd pollution may reach its farms from rain-off drift, shipping, industrial pollutants, transportation, domestic sewage, storm-water, atmospheric deposition, and construction materials among others (Al-Halani et al. 2021, 2022; Monier et al. 2023). It was hypothesized that feeding gilthead seabream on yeast-enriched diets could enhance its growth and welfare status as well as improve its performance against possible Cd pollution. According to Abdel-Tawwab et al. (2010), dietary yeast interacted with copper toxicity and minimized its effects on the performance and welfare of Galilee tilapia (*Sarotherodon galilaeus*). Therefore, the present research was done to determine the mitigation efficiency of dietary yeast (*S. cerevisiae*) against waterborne Cd toxicity effects on gilthead seabream in relation to the growth performance, haemato-biochemical, stress biomarkers, and histological investigations.

## Materials and methods

### The Cd LC_50_ determination

Gilthead seabream fingerlings (20–24 g) were purchased from a private fish farm in Borg-El Arab, Alexandria, Egypt, and acclimated for two weeks in indoor 250-L tanks during which fish were fed with the control basal diet (40% crude protein; CP) thrice daily and visually examined for health and activity.

Cadmium chloride (CdCl_2_. 2.5H_2_O; purity > 95%, MW: 183.32) was provided by El-Gomhouria Company for Trading Chemicals and Medical Appliances (Egypt) and used to make different Cd solutions. Fresh CdCl_2_ stock solution was generated by dissolving 10.0 g Cd/L in deionized water and kept at 4 °C for subsequent use of Cd concentrations in each treatment. For determining the Cd LC_50_, fish (20–24 g) were distributed into 24 120-L aquaria (10 fish/aquarium) and exposed to 0.0 (control), 0.2, 2, 5, 10, 20, 40, and 80 mg Cd/L in triplicates for 96 h. Aquaria were supported with artificial aeration via air pumps. Fish were fed on the control basal diet (40% crude protein; CP) thrice a day up to apparent satiety. Every two days, fish feces and other debris were siphoned and fifthly percent of water was replaced with new seawater containing the same initial Cd condition. Water quality parameters were daily monitored (Boyd 1984) and their values were as follows: water temperature, 25 ± 0.5 °C; dissolved oxygen, 6.3 ± 0.2 mg/L; pH, 8.4 ± 0.2; and photo-period 14 L:10 D. Fish were declared dead when gill opercula and body movement stopped. Dead fish were removed and counted every day to measure their mortality. The 96 h-LC_50_ of Cd was determined 20 mg/L following the equation of Behreus and Karber (1953). Accordingly, the values of Cd in this experiment were adjusted at sublethal doses of 1.0 and 2.0 mg/L.

### Diets preparation and fish husbandry

Dietary yeast (*S. cerevisiae*; B.F.P., Dock Road, Felixstowe, UK) was added to a control diet (40% CP) at 0%, 0.5%, and 1% (Table 1). During the mixing of the diet’s ingredients (30 min), 200 mL of water per each kg of diet is added. Feeds pastes were processed in a meat grinder and diets threads are dried at room temperature before being crushed (2–3 mm diameter). The experimental feeds were kept at – 4.0 ^o^C until they were used.

**Table 1:** Ingredients and proximate analysis (%; on dry matter basis) of diets containing different yeast levels (*S. cerevisiae*)

Ingredients	Yeast levels (%)		
	0.0 (Control)	0.5	1.0
Fish meal (72% CP)	25.0	25.0	25.0
Soybean meal (45.3%)	48.0	48.0	48.0
Wheat gluten	12.0	12.0	12.0
Corn meal	8.0	7.9	7.8
Corn oil	2.0	2.0	2.0
Fish oil	2.0	2.0	2.0
Vitamin premix ^a^	1.5	1.5	1.5
Mineral premix ^b^	1.5	1.5	1.5
Yeast	0	0.5	1.0
Total	100	100	100
Chemical composition (g/kg)			
Dry matter	9.15	9.17	9.13
Crude protein	45.88	45.86	45.84
Total lipids	14.18	14.38	14.52
Total ash	11.23	11.18	11.30

a Vitamin premix (per kg of premix): thiamine, 2.5 g; riboflavin, 2.5 g; pyridoxine, 2.0 g; inositol, 100.0 g; biotin, 0.3 g; pantothenic acid, 100.0 g; folic acid, 0.75 g; paraaminobenzoic acid, 2.5 g; choline, 200.0 g; nicotinic acid, 10.0 g; cyanocobalamine, 0.005 g; a-tocopherol acetate, 20.1 g; menadione, 2.0 g; retinol palmitate, 100,000 IU; cholecalciferol, 500,000 IU.

b Mineral premix (g/kg of premix): CaHPO4.2H2O, 727.2; MgCO4.7H2O, 127.5; KCl 50.0; NaCl, 60.0; FeC6H5O7.3H2O, 25.0; ZnCO3, 5.5; MnCl2.4H2O, 2.5; Cu(OAc)2·H2O, 0.785; CoCl3.6H2O, 0.477; CaIO3.6H2O, 0.295; CrCl3.6H2O, 0.128; AlCl3.6H2O, 0.54; Na2SeO3, 0.03

A 3 × 3 factorial design was used to assess the interaction between dietary yeast levels (0.0%, 0.5%, and 1.0%) and Cd levels (0.0, 1.0, and 2.0 mg/L). The acclimatized fish (20–24 g) for two weeks were randomly distributed into 27 120-L aquaria (15 fish/aquarium) to represent nine treatments in triplicate. Aquaria were supported with artificial aeration via air pumps. Fish were fed on the experimental diets at 9:00, 13:00, and 17:00 h until apparent satiety for 60 days. Fish feces were removed after 40 min of feeding, dried, and weighed. Every two days, fish feces and other debris were siphoned and fifthly percent of water replaced with new seawater containing the same initial Cd levels.

The water characteristics were checked daily during the experimental trial using HANNA Instruments, Portugal for measuring water temperature, digital oxygen (DO), and pH meters. The unionized ammonia (NH_3_) levels were determined according to the methods of Boyd (1984). All treatments had water temperature ranges of 25.5–27.3 °C, DO levels of 6.2–6.5 mg/L, NH_3_ levels of 0.16–0.24 mg/L, and pH levels of 8.2–8.4. The values of water quality parameters are within acceptable ranges for fish farming (Boyd and Tucker 1998).

### Growth and feed utilization indices

After the feeding period, fish in each aquarium were starved for one day before sampling and anesthetized with 100 mg/L of tricaine methanesulfonate (MS222; Sigma-Aldich, USA) according to Pires et al. (2017). All fish were counted and group-weighed to estimate the indices of fish performance and feed utilization using the given equations:

$$\begin{array}{l}\mathrm{Weight}\;\mathrm{gain}\;\%=100\;\lbrack\mathrm{final}\;\mathrm{weight}\;(\mathrm{FW})-\mathrm{initial}\;\mathrm{weight}\;(\mathrm{IW})\rbrack;\\\mathrm{Specific}\;\mathrm{growth}\;\mathrm{rate}\;(\mathrm{SGR};\;\%/\mathrm{day})=100\;\lbrack\mathrm{Ln}\;\mathrm{FW}\;(\mathrm g)-\mathrm{Ln}\;\mathrm{IW}\;(\mathrm g)\rbrack/60;\\\mathrm{Feed}\;\mathrm{conversion}\;\mathrm{ratio}\;(\mathrm{FCR})=\mathrm{total}\;\mathrm{dry}\;\mathrm{feed}\;\mathrm{intake}/\mathrm{weight}\;\mathrm{gain};\\\mathrm{Fish}\;\mathrm{survival}\;(\%)=100\;(\mathrm{fish}\;\mathrm{number}\;\mathrm{at}\;\mathrm{the}\;\mathrm{trial}\;\mathrm{end}/\mathrm{fish}\;\mathrm{number}\;\mathrm{at}\;\mathrm{the}\;\mathrm{trial}\;\mathrm{beginning}).\end{array}$$

### Blood and tissues sampling

The blood samples from five fish per aquarium (15 fish/treatment) were collected with a hypodermic syringe from the caudal vein and divided into two sets of Eppendorf tubes. One heparinized tubes were used for determining the hematological variables. At the same time, the second set was centrifuged at 5000 x g for 10 min at room temperature, and the obtained sera were stored at − 20^◦^C for further biochemical assays.

After collecting the blood, the anesthetized fish (MS222; 100 mg/L) were killed by the medullary section, washed with deionized water, and dissected. Gonad, hepatic, and spleen tissues were collected and weighed for determining gonad somatic index (GSI), hepato-somatic index (HSI), and spleen somatic index (SSI). Parts of gill, liver, and kidney tissues were removed and immediately stored (40% ethyl alcohol) for the histopathological study. Other parts of gill, liver, and kidneys tissues were stored at − 4 °C to assess Cd residue. Meanwhile, pieces of the liver and kidney tissues (100 mg) were taken and frozen directly in liquid nitrogen for measuring the DNA fragmentation % and determining the gene expression.

### Growth genes transcription

The whole mRNA was obtained from 50 mg of anterior kidney and liver tissues with Trizol (iNtRON Biotechnology, Inc., Korea). Nanodrop (Uv-Vis spectrophotometer Q5000/ Quawell, USA) confirmed the extracted mRNA quality and quantity. Following the manufacturer’s instructions, complementary DNA (cDNA) was synthesized using the SensiFASTTM cDNA synthesis kit (Bioline, United Kingdom). *IGF-1* (*Insulin-like Growth Factor 1*) and *GH* (*Growth Hormone*) genes primer sequences were employed and β-actin was tested for gene expression stability using GeNorm software 310 (M score = 0.21) and it was used as a housekeeping gene in the normalization procedure (Table 2). For gene expression, quantitative real-time PCR (qRT-PCR; Stratagene MX3000P) was used to measure gene expression using the SYBR green technique (SensiFast SYBR Lo-Rox kit, Bioline). After confirming that qRT-PCR efficiency was close to 100%, gene expression data were computed following Livak and Schmittgen (2001).

**Table 2:** PCR primer sequences, accession numbers of tested genes

Gene name Gene	Abbreviation	Sequences of primers	Amplification size	Accession No.
*Insulin-like growth factor I*	*IGF-I*	F: GGCATTGGTGTGATGTCTTT R: CATATCCTGTCGGTTTGCTG	106	AY996779.2
*Growth hormone*	*GH*	F: CGTCTCTTCTCAGCCGAT R: GCTGGTCCTCCGTCTGC	131	U01301
*ß-actin*	*actb*	F: TCCTGCGGAATCCATGAGA R: GACGTCGCACTTCATGATGCT	108	X89920

### Haemato-biochemical assays

Blood analysis was carried out as previously described by Lewis et al. (2006). Red blood cells (RBC) in blood samples were counted using a Neubauer chamber and an optical microscope (400x magnification). A commercial kit and spectrophotometer at 540 nm calculated hemoglobin (Hb) concentration. Blood was centrifuged (5 min, 1400 g) in heparinized glass capillaries to calculate hematocrit (Hct). These blood parameters were utilized to calculate mean corpuscular volume (MCV, fl.), mean corpuscular hemoglobin (MCH, pg), and mean corpuscular hemoglobin concentration (MCHC, %).

Blood cortisol levels were measured by enzyme-linked immunosorbent assay (ELISA) utilizing commercial ELISA packs (Shanghai Enzyme Biotechnology Co., Ltd.) (Han et al. 2022). Blood glucose, total cholesterol (T-CHO), triglyceride (TG), total protein (TP), albumin (ALB), globulin (GLO), lactate dehydrogenase (LDH), alanine aminotransferase (ALT), aspartate aminotransferase (AST), and alkaline phosphatase (ALP) levels were measured using clinical procedures in an automated biochemical analyzer (ADVIA 2400; SIEMENS; Han et al. 2022).

### The DNA fragmentation in liver tissues

The DNA fragmentation % was measure by spectrophotometer using diphenylamine (DPA) method, according to the method of Perandones et al. (1993). Liver tissues (1 g) were homogenized in 9 mL of lyses buffer (5 mM Tris–HCl, pH 8.0, 20 mM EDTA and 0.5% Triton X-100) and centrifuged at 1500 ×g for 20 min. Pellets were resuspended in 0.5 N perchloric acid and 5.5 N perchloric acid was added to supernatant, centrifuged again at 1500 ×g for 10 min to remove proteins. Samples were heated at 90 ◦C and after cool reacted with DPA for 16–20 h at room temperature. Absorbance was measured at 600 nm using a UV-double-beam spectrophotometer. The DNA fragmentation percentage was assessed using a reagent blank and spectrophotometer. The percentage of DNA fragmentation was expressed using the following formula:

$$\mathrm{Fragmented}\;\mathrm{DNA}\%=100\;\lbrack\mathrm{fragmented}\;\mathrm{DNA}/(\mathrm{fragmented}+\mathrm{intact}\;\mathrm{DNA})\rbrack.$$

### Determining Cd concentrations in rearing water

Water samples were collected (20 cm below the water surface) in clean 1-L plastic bottles from each aquarium. Following APHA (2005) procedures, a 50-mL water sample was put in a 500-mL Taylor flask, and 0.50 ml of concentrated sulfuric acid was added and boiled to produce white fumes. Then, samples were cooled, and 1.0 ml of 60% HCLO_3_ and 5.0 ml of concentrated HNO_3_ were added and digested until clear digest was produced. Digested samples were cooled, filtered through Whatman filter paper No. 44 into a 500-mL volumetric flask, and diluted up to 100 mL using distilled water. Cd concentrations in the resulting solutions were determined using a Flame Atomic Absorption Spectrophotometer (AAS: Perkin Elmer Analyst 100) using standard Cd concentrations.

### Cd concentrations in fish organs

Each tissue sample was dried at 105 °C before being crushed into powder; then, 1.0 g of each sample was ashed in a muffle furnace at 550 ◦C for 6 h. Afterward, ash was digested in a fume room at 80 °C with a 1:5:1 mixture of concentrated nitric, sulfuric, and perchloric acids until a colorless liquid was formed. Each digested sample was diluted to 20 ml with deionized water and Cd concentrations were determined using a Flame Atomic Absorption Spectrophotometer (AAS: Perkin Elmer Analyst 100). The Cd concentrations in different fish organs were recorded using Cd standard solutions (APHA 2005).

### Histological investigations

The histological investigation was conducted according to Gewaily et al. (2020). Gill, livers, and kidney tissues were fixed in Bouin’s solution for 18–24 h. The fixed samples were dehydrated in 70%, 80%, 90%, absolute I, II, and III alcohol, cleaned with xylene and embedded in paraffin wax. Five-micron slices were cut using a Leica rotatory microtome (RM 20,352,035; Leica Microsystems, Wetzlar, Germany), mounted on slides, and stained with hematoxylin and eosin (H&E). Finally, these slides were investigated with a BX50/BXFLA microscope (Olympus, Tokyo, Japan).

### Statistical analysis

This experiment was analyzed as 3 (yeast levels) x 3 (Cd levels) two-way ANOVA, followed by a Duncan’s Multiple Range Test. Data were transformed before statistical analysis as they failed normality (Cramer Von Mises) and homoscedasticity tests (Brown- Forsythe). All statistical analysis was performed using SPSS program version 26 (SPSS, Richmond, VA, USA).

## Results

### Growth indices and growth-related genes

**Fig. 1:**
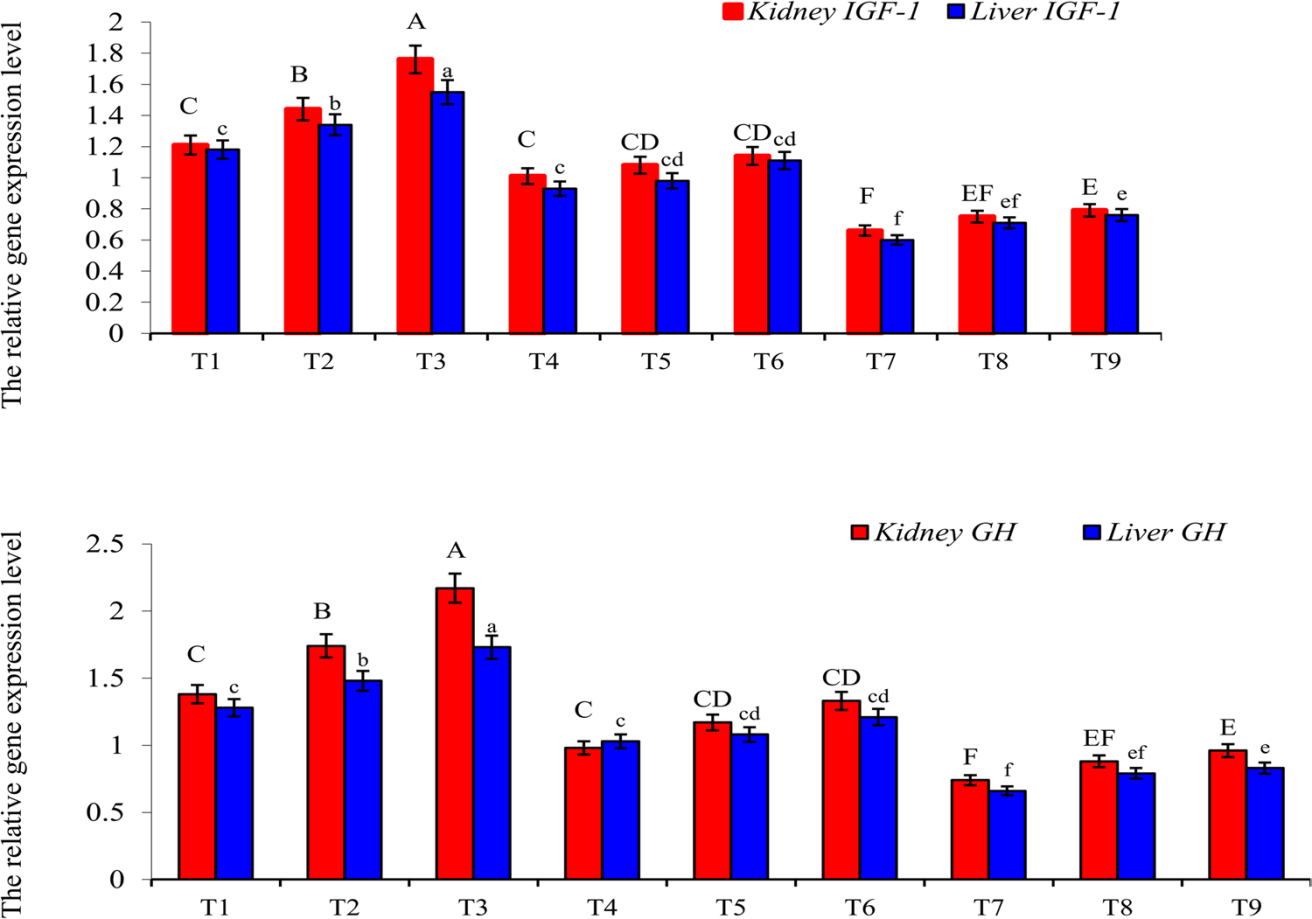
Relative expression levels of *IGF-1* and *GH* genes in the liver and anterior kidney tissues of gilthead seabream (*Sparus aurata*) fed bakery yeast (*S. cerevisiae*) along with exposure to sub-lethal levels of cadmium (Cd) toxicity for 60 days. Bars having different letters (small letters for liver and capital letters for kidney) are significantly different at *P* < 0.05. T1, 0.0 yeast + 0.0 Cd; T2, 0.5% yeast + 0.0 Cd; T3, 1.0% yeast + 0.0 Cd; T4, 0.0 yeast + 1.0 mg Cd/L; T5, 0.5% yeast + 1.0 mg Cd/L; T6, 1.0% yeast + 1.0 mg Cd/L; T7, 0.0 yeast + 2.0 mg Cd/L; T8, 0.5% yeast + 2.0 mg Cd/L; T9, 1.0% yeast + 2.0 mg Cd/L.

Growth performance indices of gilthead seabream were significantly (*P* < 0.05) affected by yeast level, Cd exposure, and their interaction (Table 3). Final weight, weight gain %, SGR, and feed intake were markedly (*P* < 0.05) increased by increasing the yeast levels, especially at the level of 1.0% (Table 3). Conversely, the growth parameters were significantly (*P* < 0.05) retarded with Cd exposure, particularly at the dose of 2.0 mg Cd/L (Table 3). In similar trends, liver and kidney expression levels of *IGF-1* and *GH* genes were considerably (*P* < 0.05) up-regulated with the increase in yeast inclusion levels in fish diets; while their expression was remarkably (*P* < 0.05) down-regulated by Cd exposure (Fig. 1). No significant (*P* > 0.05) changes were found in FCR values due to yeast levels and/or Cd exposure (Table 3). Regarding the somatic indices, GSI, HSI, and SSI values were significantly (*P* < 0.05) higher in yeast-fed fish (T1-T3), while exposing fish to Cd toxicity lowered the values of somatic indices depending on the Cd level. Fish survival rate was significantly (*P* < 0.05) lower in Cd-exposed fish, particularly at the 2.0 mg Cd/L treatments irrespective of dietary yeast levels. Feeding fish in yeast diets (T1-T3) showed higher survival with no significant (*P* > 0.05) difference among them (Table 3). It is noted that feeding Cd-exposed gilthead seabream on yeast-enriched diets, particularly at the 1.0% level recovered the adverse effects of Cd toxicity regarding the growth performance indices to be close to those of the control fish group.

**Table 3:** Growth performance and feed utilization of gilthead seabream (*Sparus aurata*) fed on yeast (*S. cerevisiae*) along with exposure to sub-lethal levels of cadmium (Cd) toxicity for 60 days

Yeast levels (%)	Cd levels (mg/L)		Initial weight (g)	Final weight (g)	Weight gain %	SGR (%/day)	Feed intake (g feed/fish)	FCR	GSI	HSI	SSI	Fish survival (%)
0.0	0	T1	22.3	50.3 c	125.6 c	1.356 c	50.8 c	1.81	0.33 ab	1.61 b	0.81 bc	100.0 a
0.5	0	T2	22.2	61.3 b	176.1 b	1.693 b	72.5 b	1.85	0.36 a	1.69 ab	0.83 b	100.0 a
1.0	0	T3	22.5	71.9 a	219.6 a	1.936 a	89.8 a	1.82	0.36 a	1.79 a	1.11 a	100.0 a
0.0	1.0	T4	22.3	41.9 e	87.9 e	1.051 d	36.1 ef	1.84	0.24 d	1.30 de	0.61 e	81.7 c
0.5	1.0	T5	22.9	43.9 de	91.7 e	1.085 d	38.5 de	1.83	0.26 cd	1.40 d	0.66 de	91.7 b
1.0	1.0	T6	22.2	48.5 cd	118.5 cd	1.302 c	49.1 cd	1.87	0.29 bc	1.50 c	0.71 cd	93.3 ab
0.0	2.0	T7	23.0	34.7 f	50.9 g	0.685 e	21.7 g	1.85	0.18 f	1.11 f	0.45 h	73.3 d
0.5	2.0	T8	23.0	38.8 ef	68.7 f	0.872 de	28.8 fg	1.82	0.19 ef	1.18 f	0.54 gh	83.3 c
1.0	2.0	T9	23.0	42.5 e	84.8 e	1.023 d	35.8 f	1.84	0.22 de	1.21 ef	0.57 fg	91.7 b
Pooled SE			0.155	2.184	10.14	0.074	4.11	0.144	0.013	0.045	0.037	1.778
Two-way ANOVA			*P* value									
Yeast levels			0.932	< 0.001	< 0.001	< 0.001	< 0.001	0.994	< 0.001	< 0.001	< 0.001	< 0.001
Cd exposure			0.252	< 0.001	< 0.001	< 0.001	< 0.001	0.966	< 0.001	< 0.001	< 0.001	< 0.001
Yeast x Cd exposure			0.864	< 0.001	0.001	0.008	0.002	0.998	0.047	0.016	0.001	< 0.001

Means followed by different letters in the same column are significantly different at *P* < 0.05 (Tukey test)

### Haemato-biochemical profile

Table 4 exhibits that WBCs, RBCs, Hb, and Hct values were significantly (*P* < 0.05) affected by dietary yeast, Cd levels, and their interaction (Table 4). MCV was significantly (*P* < 0.05) affected only by dietary yeast levels, MCHC was significantly (*P* < 0.05) affected only by Cd exposure, while MCH was significantly (*P* < 0.05) affected by both dietary yeast and Cd exposure only with no interaction (*P* > 0.05) among them (Table 4). In this regard, WBCs, RBCs, Hb, and Hct values substantially (*P* < 0.05) increased with the gradual increase in yeast levels in fish diets (T1-T3) and the decrease in Cd levels. Conversely, MCV and MCH levels considerably (*P* < 0.05) decreased by increasing dietary yeast levels and reducing the Cd levels in the case of MCH. Furthermore, MCHC is only affected by Cd exposure as it significantly increased (*P* < 0.05) by the gradual increase in Cd levels (Table 4).

**Table 4:** Hematological parameters of gilthead seabream (*Sparus aurata*) fed on yeast (*S. cerevisiae*) along with exposure to sub-lethal levels of cadmium (Cd) toxicity for 60 days

Yeast levels (%)	Cd levels (mg/L)		WBCs (×10^3^ µL)	RBCs (×10^6^ µL)	Hemoglobin (g/dL)	Hematocrit (%)	MCV	MCH	MCHC
0.0	0	T1	14.8 c	2.26 c	12.1 bc	32.5 bc	143.8 ab	53.5 bc	37.2 b
0.5	0	T2	17.8 b	2.71 ab	13.4 b	33.2 b	122.5 bcd	49.4 c	40.4 b
1.0	0	T3	22.6 a	3.07 a	14.5 a	36.3 a	118.2 cd	47.2 cd	39.9 b
0.0	1.0	T4	12.3 de	2.01 cd	10.2 d	28.6 d	142.3 abc	50.7 bc	35.7 b
0.5	1.0	T5	13.8 d	2.41 bc	12.0 bc	30.4 cd	126.1 bcd	49.8 c	39.5 b
1.0	1.0	T6	14.6 c	2.95 a	12.6 b	33.0 b	111.9	42.7 d	38.2 b
0.0	2.0	T7	11.8 e	1.37 e	9.8 d	20.7 f	151.1 a	71.5 a	47.3 a
0.5	2.0	T8	12.8 de	1.97 d	10.8 cd	23.0 ef	116.8 d	54.8 b	47.0 a
1.0	2.0	T9	13.2 d	2.28 bc	12.1 bc	24.5 e	107.5 d	53.1 bc	49.4 a
Pooled SE			0.639	0.093	0.274	0.991	2.367	1.232	0.998
Two-way ANOVA			*P* value						
Yeast levels			< 0.001	< 0.001	< 0.001	< 0.001	< 0.001	0.011	0.114
Cd exposure			< 0.001	< 0.001	< 0.001	< 0.001	0.116	< 0.001	< 0.001
Yeast x Cd exposure			< 0.001	0.045	0.048	0.045	0.500	0.386	0.621

Means followed by different letters in the same column are significantly different at *P* < 0.05 (Tukey test)

Tables 5 and 6 show that blood cortisol, glucose, T-CHO, TG, LDH, ALT, AST, and ALP were markedly (*P* < 0.05) affected by dietary yeast levels, Cd levels, and their interaction. Feeding fish on yeast-containing diets lonely (T2 - T3) significantly (*P* < 0.05) down-regulated blood cortisol, glucose, T-CHO, and TG levels; while they were up-regulating at high Cd toxicity levels (Table 5). On the other hand, LDH, ALT, AST, and ALP levels increased considerably (*P* < 0.05) with increasing Cd levels, especially at the treatment of 2.0 mg/L but feeding fish on yeast levels (T1-T3) lowered their levels (Table 6). In a similar trend, DNA fragment % was significantly affected by dietary yeast levels, Cd exposure, and their interaction (*P* < 0.05; Table 6). In Cd-free groups (T1-T3), no significant changes in DNA fragments% were observed; but under Cd exposure, feeding Cd-exposed fish on yeast considerably (*P* < 0.05) restored the DNA fragments %, particularly at the dose of 1.0% yeast (Table 6). Feeding Cd-exposed fish on yeast-enriched diets particularly at the 1.0% level restored the adverse effects of Cd toxicity in regard to the haemato-biochemical indices.

**Table 5:** Changes in blood cortisol, glucose, total cholesterol (T-CHO), and triglyceride (TG) of gilthead seabream (*Sparus aurata*) fed on yeast (*S. cerevisiae*) along with exposure to sub-lethal levels of cadmium (Cd) toxicity for 60 days

Yeast levels (%)	Cd levels (mg/L)		Cortisol (mg/dL)	Glucose (mg/dL)	T-CHO (mg/L)	TG (mg/L)
0.0	0	T1	98.3 f	55.1 e	243.6 f	270.6 d
0.5	0	T2	87.7 fg	51.3 e	223.9 g	261.7 de
1.0	0	T3	70.6 g	49.5 e	208.4 g	239.0 e
0.0	1.0	T4	257.1 e	65.1 cd	432.7 c	324.9 c
0.5	1.0	T5	235.7 d	57.2 de	4.11.0 d	309.1 c
1.0	1.0	T6	212.9 c	53.6 e	378.1 e	267.8 d
0.0	2.0	T7	716.3 a	98.3 a	487.4 a	417.0 a
0.5	2.0	T8	685.3 b	84.9 b	458.5 b	386.5 b
1.0	2.0	T9	664.0 b	72.3 c	437.6 c	366.2 b
Pooled SE			50.31	3.193	20.18	11.61
Two-way ANOVA			*P* value			
Yeast levels			0.001	< 0.001	< 0.001	< 0.001
Cd exposure			< 0.001	0.001	< 0.001	< 0.001
Yeast x Cd exposure			0.013	0.007	0.016	0.045

Means followed by different letters in the same column are significantly different at *P* < 0.05 (Tukey test)

**Table 6:** Changes in serum lactate dehydrogenase (LDH), alanine transaminase (ALT), aspartate transaminase (AST), alkaline phosphatase (ALP), and DNA fragments of gilthead seabream (*Sparus aurata*) fed yeast (*S. cerevisiae*) along with exposure to sub-lethal levels of cadmium (Cd) toxicity for 60 days

Yeast levels (%)	Cd levels (mg/L)		LDH (mg/L)	ALT (IU/L)	AST (IU/L)	ALP (IU/L)	DNA fragments (%)
0.0	0	T1	3.12 ef	16.4 cd	29.8 de	2.53 e	5.23 g
0.5	0	T2	2.84 f	15.5 d	26.7 ef	2.31 ef	4.31 g
1.0	0	T3	2.08 g	13.3 e	23.5 f	2.12 f	3.95 g
0.0	1.0	T4	3.92 d	17.8 c	36.5 b	4.30 c	16.37 d
0.5	1.0	T5	3.50 de	16.6 cd	32.7 cd	3.86 c	11.59 e
1.0	1.0	T6	3.23 ef	14.8 de	30.9 d	3.16 d	8.36 f
0.0	2.0	T7	5.95 a	23.1 a	41.6 a	5.97 a	36.64 a
0.5	2.0	T8	5.06 b	20.6 b	38.1 ab	5.53 ab	31.14 b
1.0	2.0	T9	4.51 c	18.3 c	35.4 bc	5.02 b	20.83 c
Pooled SE			0.224	0.571	1.067	0.268	2.389
Two-way ANOVA			*P* value				
Yeast levels			< 0.001	< 0.001	< 0.001	< 0.001	< 0.001
Cd exposure			< 0.001	< 0.001	< 0.001	< 0.001	< 0.001
Yeast x Cd exposure			0.026	0.223	0.929	0.104	< 0.001

Means followed by different letters in the same column are significantly different at *P* < 0.05 (Tukey test)

### Deposited Cd in fish organs

The Cd levels in rearing water during the experimental trial were affected only by Cd exposure levels (*P* < 0.05; Table 7) where it was 0.004–0.005 mg/L in Cd-free treatments (T1-T3); meanwhile it reached 0.88–0.90 mg/L in T4-T6 treatments. The highest Cd levels in aquaria water were observed in T7-T9 treatments (1.89–1.91 mg/L; Table 7). The Cd concentrations in different tissues of gilthead seabream are significantly (*P* < 0.05) affected by yeast levels, Cd levels, and their interaction (Table 7). The Cd residues in gill, liver, and kidney tissues were significantly (*P* < 0.05) higher in Cd-exposed fish particularly at 2.0 mg Cd/L treatments irrespective of yeast levels; meanwhile lowest Cd residues were significantly (*P* < 0.05) detected in yeast-fed fish lonely with no Cd exposure (T2-T3) showing no significant (*P* > 0.05) differences among them (Table 7).

**Table 7:** Changes in cadmium (Cd) concentration in rearing water as well as gills, liver, and muscles tissue of gilthead seabream (*Sparus aurata*) fed on yeast (*S. cerevisiae*) along with exposure to sub-lethal levels of cadmium (Cd) toxicity for 60 days

Yeast levels (%)	Cd levels (mg/L)		Cd in rearing water (mg/L)	Cd in gills (mg/g fresh weight)	Cd in liver (mg/g fresh weight)	Cd in muscles (mg/g fresh weight)
0.0	0	T1	0.005 c	0.016 g	0.027 c	0.015 g
0.5	0	T2	0.005 c	0.015 g	0.026 c	0.014 g
1.0	0	T3	0.004 c	0.014 g	0.022 c	0.013 g
0.0	1.0	T4	0.88 b	6.14 d	11.23 d	0.811 d
0.5	1.0	T5	0.90 b	4.96 e	9.60 e	0.536 e
1.0	1.0	T6	0.88 b	3.35 f	5.87 f	0.394 f
0.0	2.0	T7	1.91 a	12.48 a	22.40 a	1.351 a
0.5	2.0	T8	1.89 a	10.21 b	18.33 b	1.189 b
1.0	2.0	T9	1.89 a	8.23 c	16.90 c	0.970 c
Pooled SE			0.152	0.8772	1.565	0.0964
Two-way ANOVA			*P* value			
Yeast levels			0.996	< 0.001	< 0.001	< 0.001
Cd exposure			< 0.001	< 0.001	< 0.001	< 0.001
Yeast x Cd exposure			0.989	< 0.001	< 0.001	< 0.001

Means followed by different letters in the same column are significantly different at *P* < 0.05

### Histopathological investigations

**Fig. 2:**
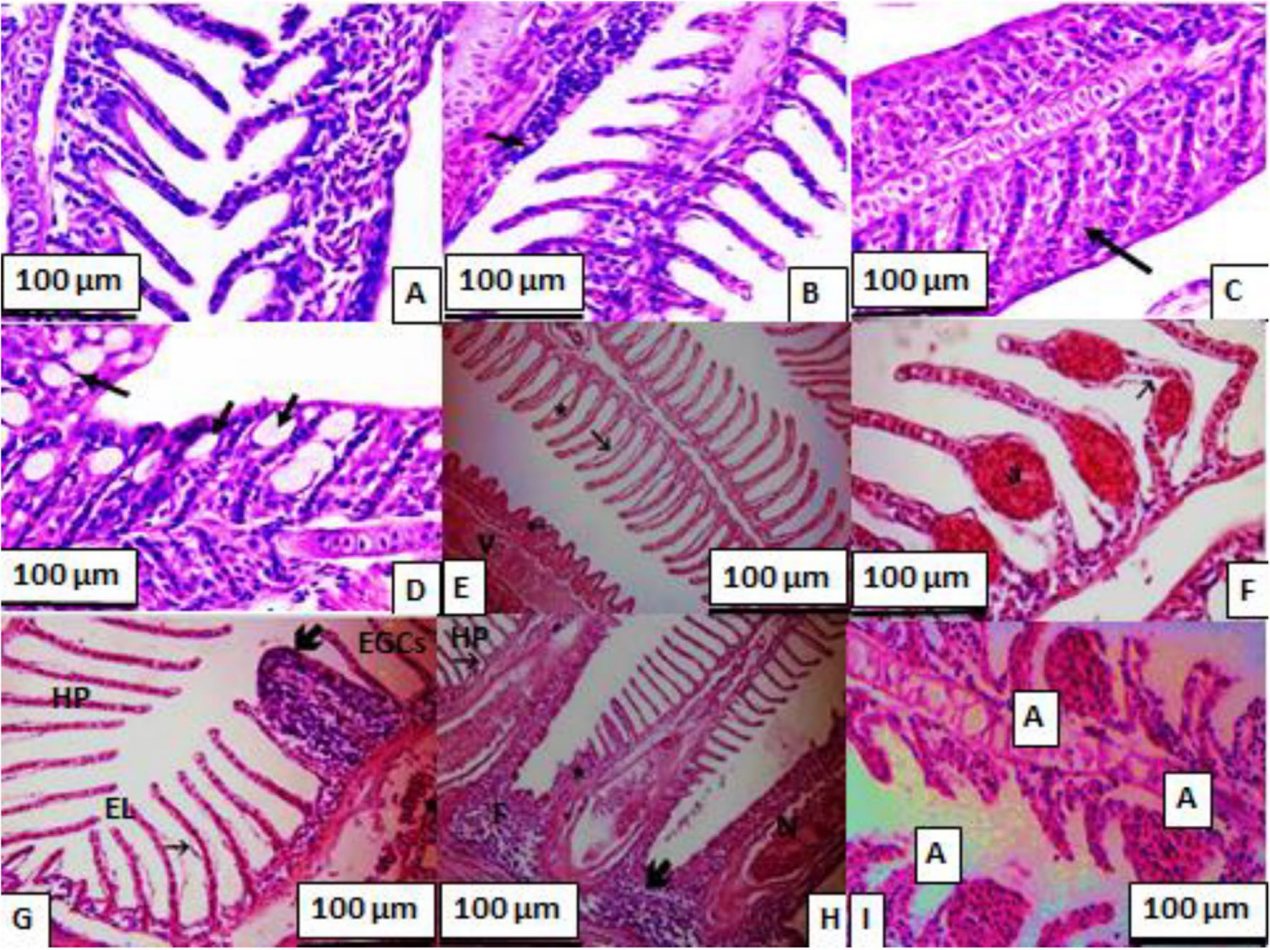
Gill sections of gilthead seabream; **A** 0.0 yeast levels + 0.0 Cd levels (control); showing normal gill filament and gill arch; **B** 0.5% yeast level + 0.0 Cd levels; showing epithelial cells on secondary lamellae; leukocytes; an increase of mucous cells; chloride cells (arrow); **C** 1.0% yeast levels + 0.0 Cd levels; showing activation of goblet and increase of leucocytic cells (arrow); **D** 0.0 yeast level + 1.0 mg Cd/L; showing hypertrophy of the lamellae epithelium end (arrows); **E** 0.5% yeast level + 1.0 mg Cd/L; showing separation of surface epithelium from capillary beds by edema (* & arrows) and focal fusion of secondary gill lamellae (V & arrow); **F** 1.0% yeast levels + 1.0 mg Cd/L; showing sever dilatation of blood vessels (Telangectasis) (a & arrow); **G** 0.0 yeast level + 2.0 mg Cd/L; showing eosinophilic granular cells (EGCs) infiltration, epithelial lifting (EL), hyperplasia of the epithelium (HP) (arrows); **H** 0.5% yeast level + 2.0 mg Cd/L; showing edema in the filamentary epithelium, dilation of the central venous with blood congestion, hyperplasia of the epithelium (HP), a fusion of the secondary lamellae (F) and necrosis (N) in primary and secondary lamellae, and **I** 1.0% yeast level + 2.0 mg Cd/L; showing complete lysis of lamellae epithelium and heavy leukocytes infiltration, hypertrophy of the lamellae epithelium end with edema (A), and hyperplasia of interlamellae (H&E.; x160)

**Fig. 3:**
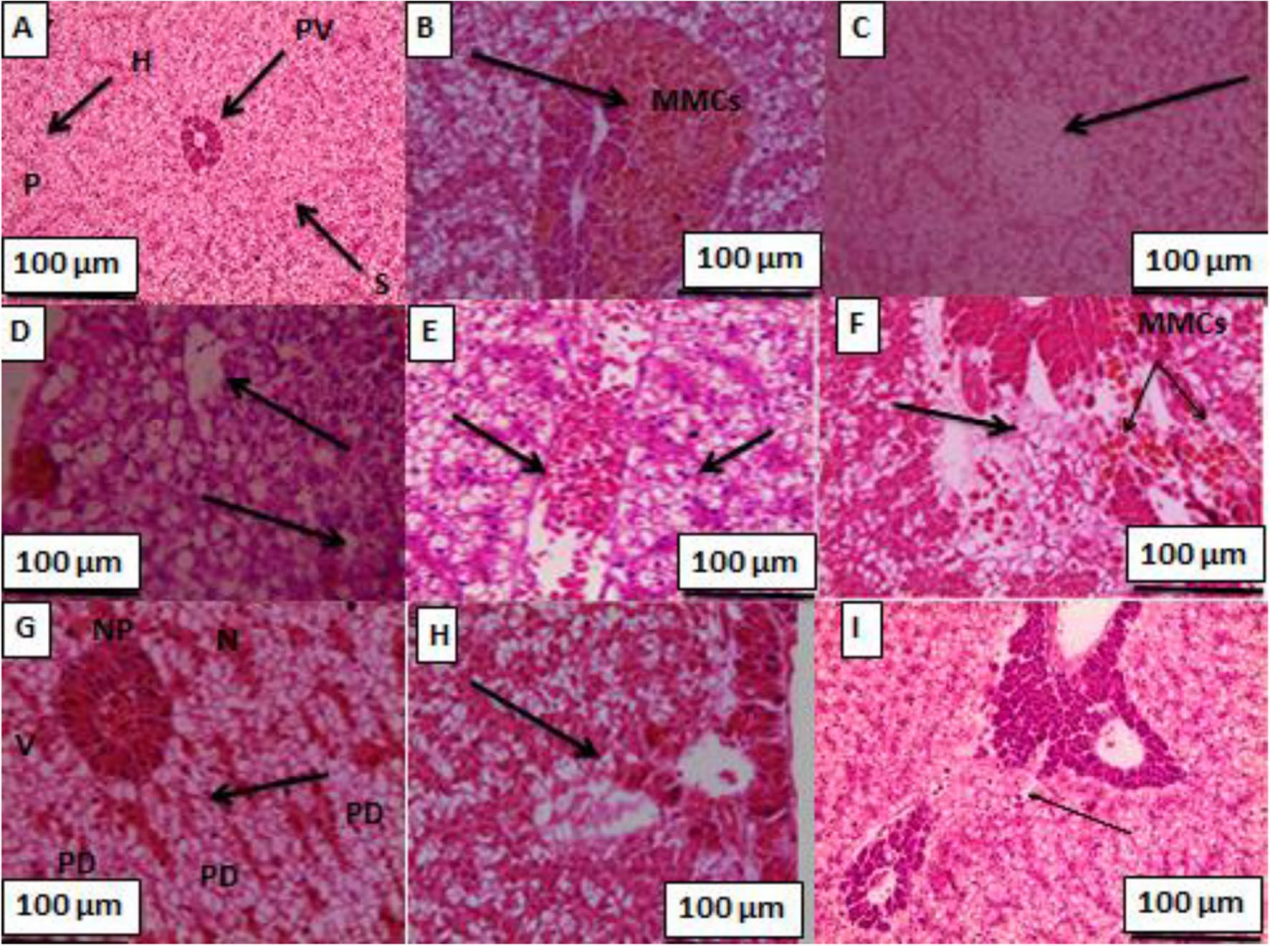
Liver sections of gilthead seabream; **A** 0.0 yeast + 0.0 Cd level (control); showing a normal architecture of hepatic cells (H), sinusoids (S), portal vein (PV) with pancreatic tissue (P); **B** 0.5% yeast + 0.0 Cd level; showing activation of melanomacrophage centers (MMCs)(arrow); **C** 1.0% yeast + 0.0 Cd level; showing homogeneous parenchyma tissues (arrow); (D) 0.0 yeast + 1.0 mg Cd/L; showing steatosis vacuolation and necrosis (arrows); **E** 0.5% yeast + 1.0 mg Cd/L; showing swelling of hepatocytes and congested blood vessels (arrows); **F** 1.0% yeast + 1.0 mg Cd/L; showing slight necrosis of hepatic tissue with activation of MMCs (arrows); **G** 0.0 yeast level + 2.0 mg Cd/L; showing multifocal areas of necrosis in hepatic and pancreatic tissue, patchy degeneration (PD),cytoplasmic vacuolation (V), necrosis (N) with nuclear piknosis (NP) (arrow); **H** 0.5% yeast + 2.0 mg Cd/L; showing wide area of necrosis in hepatopancreas, and **I** 1.0% yeast + 2.0 mg Cd/L; showing an incresee number of pancreatic acenia and focal area of necrosis and lyses of hepatic tissue (arrow) (H&E; x 160)

**Fig. 4:**
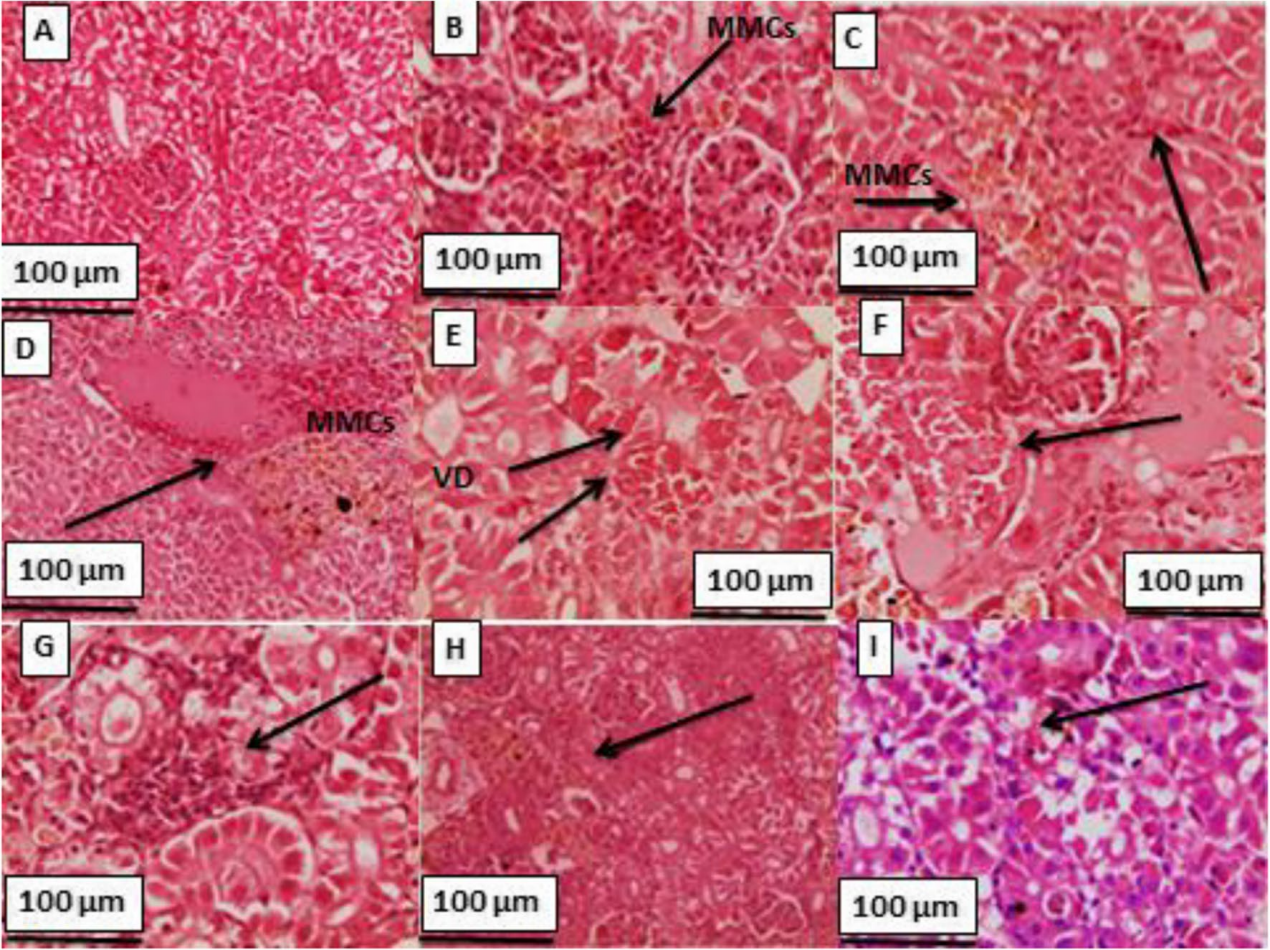
Kidney sections of gilthead seabream; **A** 0.0 yeast levels + 0.0 Cd; showing normal renal tissue architecture; **B** 0.5% yeast levels + 0.0 Cd; showing activation of MMCs (arrow); **C** 1.0% yeast + 0.0 Cd; showing activation of MMCs (arrow); **D** 0.0 yeast level + 1.0 mg Cd/L; showing vacuolar degeneration of tubular epithelium, atrophy of glomerular tuft and slight activation of MMCs (arrows); **E** 0.5% yeast + 1.0 mg Cd/L; showing vascular degeneration of tubular epithelium (VD), mononuclear cell infiltration between renal tubules (arrows); **F** 1.0% yeast levels + 1.0 mg Cd/L; showing normal renal tissue with slight congestion and hemorrhage; **G** 0.0 yeast + 2.0 mg Cd/L; showing sever hemorrhage, damage and necrosis of renal tissue (arrow); **H** 0.5% yeast + 2.0 mg Cd/L; showing hemorrhages, degeneration and necrosis of tubular epithelium, and **I** 1.0% yeast + 2.0 mg Cd/L; showing focal area of necrosis, with multiple hemorrhagic foci (arrow) (H&E; x 160)

The histopathological examination of the control group (T1) revealed the normal structure of the gill, liver, and kidney tissues (Figs. 2, 3, and 4). The histopathological changes in gill tissues of gilthead seabream fed on different levels of yeast along with Cd exposure are presented in Fig. 2. The fish fed on the control diet (0.0 yeast level + 0.0 Cd level) show regular gill filament and gill arch; meanwhile the gill of yeast-fed fish (T1-T3) show epithelial cells on secondary lamellae, activation of goblet, and increases of leucocytic cells. Conversely, exposing gilthead seabream to Cd toxicity only shows eosinophilic granular cells (EGCs) infiltration, epithelial lifting (EL), and hyperplasia of the epithelium (HP). Feeding fish on yeast levels especially at the high level (1.0%) restored the above-mentioned features in the architecture of gill tissues.

Figure 3 shows that fish fed on the control diet (T1) show normal architecture of hepatic cells (H), sinusoids (S), and portal vein (PV) with pancreatic tissue (P). feeding fish on yeast-enriched diets lonely (T2-T3) shows activation of melanomacrophage centers (MMCs) and homogeneous parenchyma tissues. On the other hand, exposing gilthead seabream to Cd toxicity causes steatosis vacuolation and multifocal areas of necrosis in hepatic and pancreatic tissue, patchy degeneration (PD), cytoplasmic vacuolation (V), necrosis (N) with nuclear pyknosis (NP). Feeding fish on yeast-containing diets restored the damage that occurred in hepatic cells due to Cd toxicity showing light necrosis of hepatic tissue with activation of MMCs.

The histopathological changes in kidney tissues of gilthead seabream fed on different yeast levels along with Cd exposure levels are presented in Fig. 3. The fish fed on the control diet with no Cd exposure (T1) show normal renal tissue architecture; meanwhile feeding fish on yeast diets only (T2-T3) show marked activation of MMCs. On the other hand, fish exposed to Cd toxicity only show vacuolar degeneration of tubular epithelium, atrophy of glomerular tuft, severe hemorrhage, damage, and necrosis of renal tissue (Fig. 3G). The co-feeding of gilthead seabream along with Cd exposure recovered the Cd adverse effects showing normal renal tissue with slight congestion and hemorrhage (Fig. 3H-I).

## Discussion

### Growth indices and growth-related genes

In the present study, dietary yeast increased the growth performance of gilthead seabream. This may be because yeast cells contain many bioactive components that exhibited positive effects on fish growth and welfare status (Hansen et al. 2019; Rawling et al. 2019; Vidakovic et al. 2020). The enhanced growth in yeast-fed fish may be because yeast prompted the feed palatability resulting in enhancing the feed intake. Additionally, it plays a positive role in increasing the gastrointestinal microbiota (Huyben et al. 2017; Xia et al. 2022) and promoting the development of digestive enzymes (Castro et al. 2013), which assist in feed digestion and nutrients utilization providing the fish with more enzymes, minerals, vitamins, and amino acids among others.

Growth is a polygenic and environmentally regulated feature, with *IGF-1* and *GH* that being the most significant growth genes (Triantaphyllopoulos et al. 2020). IGF-1 production is influenced by insulin, growth hormone, other hormones, and metabolic and dietary circumstances (Larsen et al. 2001). Therefore, nutrients may play a pivotal role in regulating *IGF-1* and *GH* genes expression. In the present investigation, feeding gilthead seabream on *S. cerevisiae* resulted in a substantial upregulation in the expression of *IGF-1* and *GH* genes. Several studies have found the positive effect of dietary yeast (*S. cerevisiae*) on the growth indices of common carp, *Cyprinus carpio* (Manoppo and Kolopita 2016) and Nile tilapia, *Oreochromis niloticus* (Abdel-Tawwab 2012; Abdel-Tawwab et al. 2008, 2020), European seabass, *Dicentrarchus labrax* (Korni et al. 2021), rainbow trout, *Oncorhynchus mykiss* (Vidakovic et al. 2020), and gilthead seabream (Dawood et al. 2017; Fath El-Bab et al. 2022).

Contrarily, exposing the fish to waterborne Cd, in the current study, retarded their performance as compared with the control group. These results may be linked to the down-regulation of *IGF-1* and *GH* genes expression in Cd-exposed fish. The Cd’s toxicity increased metabolic needs and disrupting normal physiological functions leading to growth restriction (Hogstrand et al. 1996). Waiwood and Beamish (1978) found that Cd exposure affects salmonids’ basal metabolism, limiting its development via lower energy efficiency and higher metabolic maintenance costs. In our study herein, the growth inhibition in the Cd-exposed fish may be owing to its deteriorating effects on feed intake and nutrients absorption. previous studies reported that exposing fish to Cd beneath its lethal levels have been observed to inhibit the growth indices in rainbow trout (Ricard et al. 1998), juvenile bull trout, *Salvelinus confluentus* (Hansen et al. 2002), Nile tilapia (Abdel-Tawwab and Wafeek 2010, 2017; Elgendy et al. 2023), and guppy, *Poecilia reticulate* (Miliou et al. 1998).

Feeding the Cd-exposed fish on dietary yeast (*S. cerevisiae*), in the current study, restored their restricted growth to be near those of the control group. These results evoked that dietary yeast played a crucial role in alleviating the toxic effects of waterborne Cd via chelating the Cd metal and/or accelerating its release into the surrounding ecosystem. In a similar study, Abdel-Tawwab et al. (2010) reported that dietary yeast was efficient in alleviating the toxic effects of waterborne copper on the growth indices of Galilee tilapia. In a similar study, Tao et al. (2021) stated that dietary yeast culture improved CCl4-induced liver damage and inflammatory response via inhibition of TLR2/NF-kB signaling pathway expression in *Pseudobagrus ussuriensis*. Elgendy et al. (2023) found that dietary onion (*Allium cepa*) enhanced the growth of Cd-exposed Nile tilapia.

### Haemato-biochemical profile

Blood parameters are important indicators of fish health because they reflect stresses and exterior stimuli as well as the nutritional status of fish. In this research, a dose-dependent increase in dietary yeast promoted WBCs, RBCs, Hb, and Hct values, whereas they were lower at Cd-intoxicated fish. The blood values will indicate optimal nutrient consumption and growth rate. An accurate fish haemogram analysis revealed a considerable rise in RBCs, hemoglobin, and hematocrit content in all yeast-fed fish groups. This rise in haemogram readings is often connected to yeast bioactive compounds that played a role in enhancing the hematopoiesis process (Arup and Patra 2011; Rajesh et al. 2006). The total WBCs count of yeast-treated fish increased significantly compared to the control group. Sang and Fotedar (2010) obtained similar findings, which may be attributable to the yeast cell wall components, namely β-glucan, which have unique receptors for phagocytic cells (heterophile and monocytes). β-Glucan binds to the surface receptor molecules of circulating and tissue phagocytes. This binding will boost the phagocytic activities of bacteria ingestion, destruction, and digestion. Simultaneously, they emit signal molecules (cytokines) that promote the development of new WBCs. On the other hand, the negative impacts of Cd on hematological parameters could be due to abnormalities in the hemopoietic and metabolic status of the Cd-treated fish. In this regard, Sharma and Langer (2014) stated that exposing fish to sublethal Cd levels caused hemopoietic organ malfunction, resulting in low Hb concentrations.

In yeast, *S. cerevisiae*-supplemented groups, in the present research, lower levels of serum T-CHO and TG were substantially detected suggesting the favorable impact of dietary yeast, *S. cerevisiae*, on lipid profile. Meanwhile, higher T-CHO and TG levels observed with Cd toxicity might be attributed to renal tissue injury. In this regard, Ayiku et al. (2020) showed substantial reductions in serum T-CHO and TG levels in shrimp given diets containing 2% yeast. Fath El-Bab et al. (2022) concluded that serum T-CHO and TG levels were reduced in the yeast-fed sea bream group compared to the control one.

Heavy metals including Cd could cause malfunctions in many fish tissues; thus, enzymes bioassay may measure tissues damage induced by heavy metals exposure (Abdel-Tawwab and Wafeek 2010, 2017). ALT and AST play a key role in amino acids and protein metabolism and may be released into the plasma after tissue injury or malfunction. In the current study, high levels of serum LDH, AST, and ALT, and ALP values in Cd-intoxicated fish suggest the existence of a cytoplasmic enzyme discharged into the blood following liver injury (Bernet et al. 2001; Yousefi et al. 2020). El-Naga et al. (2005) found that Cd exposure increased AST and ALT values in marine fish (*Mugil seheli*). Thirumavalavan (2010) found that AST and ALT activity increased in *Oreochromis mossambicus* tissues exposed to Cd for 7 and 14 days owing to necrosis and increased cell membrane permeability, causing tissue damage. On the other hand, fish fed on yeast-supplemented feeds had lower values of serum LDH, AST, ALT, and ALP than the control fish group, implying that aquatic animal diets may benefit from adding yeast to fish diets (Hassaan et al. 2018).

### Stress biomarkers

Cortisol and glucose levels are major characteristic and useful stress bioindicators in fish (Barton and Iwama 1991). Usually, chronic stress may cause physiological changes in cortisol and glucose levels (McEwen 2008). The current research indicates higher levels of cortisol and glucose in Cd-intoxicated fish groups. These results may be due to glycogenolysis, releasing the glucose from glycogen in muscles and the liver, triggered by stress hormones (cortisol and catecholamines). Similarly, blood glucose levels in Cd-exposed common carp (Cicik and Engin 2005) and Cd-exposed Nile tilapia (Abdel-Tawwab and Wafeek 2017) were higher than the control group. On the other hand, dietary yeast in the present study lowered glucose levels; this may be due to the presence of β-glucan, one of the yeast components, which increases intestinal viscosity resulting in slow glucose absorption from the bloodstream (Pilarski et al. 2017; Sánchez-Martínez et al. 2017). Similar results were found by Abdel-Tawwab et al. (2010) who stated that feeding Galilee tilapia on yeast reduced the blood glucose level in copper exposed fish.

### Deposited Cd in fish organs

Feeding gilthead seabream on yeast-enriched diets in the current study showed no significant differences in Cd residue in gill, liver, and muscle tissues; meanwhile the Cd residues in these tissues were higher in Cd-intoxicated fish as compared with the control fish group. The administration of yeast to Cd-exposed fish pointedly lowered Cd residue (T4 vs. T5-T6 and T7 vs. T8-T9. Cadmium dissolved in water [in the form of Cd (II) ions] was immediately absorbed by aquatic organisms through the absorption process (Idrees et al. 2020). Cadmium is quickly absorbed by fish gill and skin by passive diffusion (Okocha and Adedeji 2011). Cadmium ions entered the gill via calcium channels owing to the high affinity of Cd^2+^ for Ca^2+^ binding sites (Flik et al. 1985). The deposition of Cd in fish liver was higher than that in gill but the lowest Cd residue was observed in muscle tissues. These results are much more expected where liver tissues are the site of natural binding proteins in hepatic tissues such as metallothionein that is responsible for metals chelating, metabolism, and detoxification (Görür et al. 2012; Abdel-Tawwab and Wafeek 2014). The low Cd accumulation levels in fish muscles is because muscle is not an active tissue in accumulating heavy metals (Bahnasawy et al. 2009). In this regard, Al-Halani et al. (2021) and Al-Halani et al. (2022) found that highest Cd residue was observed in liver tissues, while it was the lowest in muscle tissues of European seabass and common sole fish, *Solea solea*, respectively. Monier et al. (2023) found that highest Cd residue was observed in liver tissues, while it was the lowest in muscle tissues of grey mullet (*Mugil cephalus*), red seabream (*Pagrus pagrus*), and sardine (*Sardinella aruita*).

On the other hand, the decrease in Cd accumulation levels in different organs of Cd-exposed fish along with increasing dietary yeast levels is because the yeast cell wall initially contacts excess cations in heavy metals-contaminated conditions. If the Cd pollution is not extreme, the cations would likely remain at this level owing to the highly phosphorylated and carboxylated mannoproteins (together with β-glucan and chitin) that decorate the cell facade with a negatively charged shield susceptible to attach to positively charged species, such as the metal cations (Cabib and Arroyo 2013). Excess metal ions that escape the negatively charged groups on the cell wall surface enter the porous cell wall and reach the membrane to damage the lipid bilayer or membrane transporters (Farcasanu et al. 2018). Furthermore, the rigid cell-wall of fungi (as yeast) is made up of chitin, inorganic ions, lipids, nitrogen-containing polysaccharide, polyphosphates, and proteins (Agboola et al. 2021; Ceseña et al. 2021) that could chelate with metals ions, extracellular and intracellular precipitation, and valence transformation, with many absorbing heavy metals into their mycelium and spores resulting in the removal of metals (Gupta et al. 2015). Elgendy et al. (2023) found that dietary onion reduced the Cd accumulation in organs of Nile tilapia.

### Histopathological investigations

Histopathological investigations could be used as indicators for the assessment of the toxic effects of heavy metals including Cd toxicity on aquatic organisms. The histological examination is crucial in determining cellular changes that may occur in target organs, such as gill, liver, and kidney tissues. As a result of the exposing gilthead seabream to waterborne Cd, in this study, significant histological changes were observed in gill, liver, and kidney tissues. In this regard, Thophon et al. (2003) stated that Cd caused histological changes in kidney, gills, liver and gastrointestinal tract, anemia in white seabass, *Lates calcarifer*. Ahmed et al. (2014) observed many histological changes in gill, liver, and kidney tissues in Cd-exposed *Anabas testudineus*. They observed proliferation of epithelial cells, fusion of secondary lamellae, hyperplasia and hypertrophy of mucous cells, and necrosis of epithelial cells in gill tissues of Cd-exposed fish. The liver tissues showed darker nucleoli, irregularly shaped hepatocytes with dilated blood capillaries, and focal as well as single necrosis. Kidney tissue showed cells necrosis, degenerated kidney tubules, congestion, lymphocytic infiltration and vacuolation.

Feeding the Cd-intoxicated fish with dietary yeast restored the architecture of gill, liver, and kidney tissues, which was damaged in Cd-exposed fish lonely (T4 and T7). These results could be due to dietary yeast lowered the Cd residue in these tissues (Table 7) showing some pathological changes including moderate congestion of pancreatic acini and hepatic sinusoids with vacuoles of hepatocytes. These findings agree with those of Long et al. (2017), who found that dietary yeast, *S. cerevisiae* alleviated the histopathological effects of nitrite exposure in the liver of Wuchang bream (*Megalobrama amblycephala*). In a similar study, Ünlü et al. (2009) and Otludil et al. (2017) reported that feeding Cd-exposed Nile tilapia on green alga (*Cladophora glomerata*) accumulated more Cd in its body and lightened its adverse effects, and enhanced the gill histopathology.

Fish fed on the control diet only (T1) showed little damage in gill, liver, and kidney tissues, including degeneration and necrosis in hepatocytes. This is often occurred after feeding fish on diets with high soybean (Hassaan et al. 2018). On the other hand, microcopy observations revealed normal architecture of these organs with no damage after yeast supplementation to gilthead seabream. These investigations evoked that dietary yeast supplementation, especially at high dietary yeast levels overcame these pathological aspects and maintained the normal histo-structure (Bacha Jr and Bacha 2012). The increase in dietary *S. cerevisiae* levels was associated with the good health status and integrity of liver hepatocytes (Safdari-Rostamabad et al. 2017). Histological analysis of the liver of sea bass fed different levels of yeast extract showed steatosis with fat degeneration, while liver morphology was considerably improved with yeast extract supplementation.

## Conclusion

The present investigation was done to evaluate the interactive effect of Cd toxicity and dietary yeast on growth performance, haemato-biochemical, and stress status of gilthead seabream. It is noted that feeding gilthead seabream on dietary yeast markedly improved its performance and welfare especially at the level of 1% yeast. Conversely, exposing fish to waterborne Cd adversely affected the fish growth and deteriorated its health status. The administration of yeast to Cd-intoxicated fish restored the Cd toxicity effects and decreased its accumulation in fish tissues, normalizing tissues architecture after their Cd-induced damage.

## Data Availability

All data of this study are included in this article.
